# Diagnostic Performance of ChatGPT-5 for Detecting Pediatric Pneumothorax on Chest Radiographs: A Multi-Prompt Evaluation

**DOI:** 10.3390/diagnostics16020232

**Published:** 2026-01-11

**Authors:** Chih-Hao Wang, Po-Chih Lin, Shin-Lin Shih, Pei-Shan Tsai, Wen-Hui Huang

**Affiliations:** 1 Department of Pediatrics, MacKay Children’s Hospital, Taipei 104217, Taiwan; mmh4972@gmail.com (C.-H.W.); freedom20031023@gmail.com (P.-C.L.); 2 Department of Radiology, MacKay Memorial Hospital, Taipei 104217, Taiwan; pololololo@mail2000.com.tw; 3 School of Medicine, Taipei Medical University, Taipei 110301, Taiwan; 4 Department of Medicine, Mackay Medical University, New Taipei City 252005, Taiwan; 5 Mackay Junior College of Medicine, Nursing and Management, Taipei 112021, Taiwan; 6 Department of Biomedical Imaging and Radiological Sciences, National Yang Ming Chiao Tung University, Taipei 112304, Taiwan

**Keywords:** ChatGPT, large language model, pneumothorax, pediatric emergency medicine, chest radiograph, prompt engineering

## Abstract

**Background/Objectives**: Chest radiography is the primary first-line imaging tool for diagnosing pneumothorax in pediatric emergency settings. However, interpretation under clinical pressures such as high patient volume may lead to delayed or missed diagnosis, particularly for subtle cases. This study aimed to evaluate the diagnostic performance of ChatGPT-5, a multimodal large language model, in detecting and localizing pneumothorax on pediatric chest radiographs using multiple prompting strategies. **Methods**: In this retrospective study, 380 pediatric chest radiographs (190 pneumothorax cases and 190 matched controls) from a tertiary hospital were interpreted using ChatGPT-5 with three prompting strategies: instructional, role-based, and clinical-context. Performance metrics, including accuracy, sensitivity, specificity, and conditional side accuracy, were evaluated against an expert-adjudicated reference standard. **Results**: ChatGPT-5 achieved an overall accuracy of 0.77–0.79 and consistently high specificity (0.96–0.98) across all prompts, with stable reproducibility. However, sensitivity was limited (0.57–0.61) and substantially lower for small pneumothoraces (American College of Chest Physicians [ACCP]: 0.18–0.22; British Thoracic Society [BTS]: 0.41–0.46) than for large pneumothoraces (ACCP: 0.75–0.79; BTS: 0.85–0.88). The conditional side accuracy exceeded 0.96 when pneumothorax was correctly detected. No significant differences were observed among prompting strategies. **Conclusions**: ChatGPT-5 showed consistent but limited diagnostic performance for pediatric pneumothorax. Although the high specificity and reproducible detection of larger pneumothoraces reflect favorable performance characteristics, the unacceptably low sensitivity for subtle pneumothoraces precludes it from independent clinical interpretation and underscores the necessity of oversight by emergency clinicians.

## 1. Introduction

Pneumothorax is a potentially life-threatening condition that requires rapid recognition and timely management in the emergency department [1]. According to the American College of Radiology, any delay in notification of pneumothorax can lead to clinical deterioration and potential complications [2]. Diagnosing pneumothorax in children poses unique challenges because of anatomical differences and limited symptom expression [3,4]. Chest radiography remains the primary first-line imaging tool in pediatric emergency settings; however, image interpretation can be delayed or erroneous under the pressures of high patient volume, variable clinician experience, and fatigue [5,6]. This diagnostic challenge is particularly pronounced for small or early pneumothoraces, which may exhibit only subtle radiographic signs and are prone to underdiagnosis [7].

Recent advances in artificial intelligence (AI), particularly in deep learning models such as convolutional neural networks (CNNs), have demonstrated promising performance in medical image classification [8,9]. Yet, these systems are typically confined to specialized radiology infrastructure and have rarely been validated in pediatric or real-world emergency contexts [10,11].

In contrast, multimodal large language models (LLMs), such as ChatGPT, have rapidly emerged as accessible general-purpose tools capable of both image understanding and natural language reasoning [12]. Their “plug-and-play” convenience allows frontline clinicians to query or upload images without specialized software, effectively creating an unregulated real-world experiment in AI-assisted medical decision-making [13]. Despite their convenience, the diagnostic reliability, consistency, and limitations of such general-purpose models for specialized clinical imaging tasks remain largely unknown.

This study aimed to evaluate the diagnostic performance of ChatGPT-5 in detecting and localizing pneumothorax on pediatric chest radiographs. We systematically assessed its accuracy, sensitivity, specificity, and localization performance (laterality) across multiple prompting strategies and examined the reproducibility of its outputs upon repeated testing.

## 2. Materials and Methods

### 2.1. Ethical Statement and Informed Consent

The study was approved by the Institutional Review Board of MacKay Memorial Hospital (Taipei, Taiwan; approval no. 25MMHIS258e), and the requirement for informed consent was waived. Reporting of the study follows the Strengthening the Reporting of Observational Studies in Epidemiology (STROBE) guidelines [14].

### 2.2. Study Design and Settings

We conducted a retrospective study of data from October 2014 to June 2025 in the pediatric emergency and inpatient departments of MacKay Memorial Hospital, a two-branch tertiary medical center in northern Taiwan. All eligible chest X-rays (CXRs) were obtained from the hospital’s electronic medical records (EMR) and picture archiving and communication systems (PACS). Data extraction and processing were performed after institutional approval.

### 2.3. Study Participants

We retrospectively identified pediatric patients (<18 years of age) diagnosed with pneumothorax using International Classification of Diseases, Tenth Revision (ICD-10) codes J93. * and ICD-9 code 512.*. Two reviewers (pediatric emergency physicians) screened each candidate’s record and corresponding CXR. Patients were included if the radiology report explicitly mentioned pneumothorax and the imaging findings were consistent with the report.

The exclusion criteria were poor image quality, non–posteroanterior (PA) view radiographs, concurrent pulmonary disease (e.g., pneumonia, emphysema), visible devices or artifacts (e.g., chest tubes, pigtail catheters, clothing buttons), and incomplete clinical or imaging data.

The control group comprised pediatric PA-view CXRs interpreted as showing no significant abnormalities and randomly selected from a pool of eligible images obtained from the same clinical context and time period, after matching for age and sex with the pneumothorax group.

### 2.4. Data Collection

All CXRs were retrieved from the hospital’s PACS and processed in an anonymized JPEG format. The images, typically sized approximately 2200 × 2672 pixels, were prepared for two reasons: first, to ensure compatibility with the ChatGPT interface, and second, to emulate the image quality and specifications typically available at the point of care. Although JPEG compression inherently reduces image fidelity relative to diagnostic-grade DICOM files, no perceptible loss of resolution or grayscale detail was noted for pneumothorax identification. No additional image preprocessing (e.g., resizing, cropping, contrast enhancement, denoising, or normalization) was applied before upload.

Ground-truth labeling for both the pneumothorax and control groups was established by two board-certified radiologists (with 13 and 15 years of experience, respectively) who were blinded to the original radiology reports and patient group assignment. They independently reviewed each image to determine the presence and laterality of pneumothorax for all cases, and for those deemed positive, the size classification according to the American College of Chest Physicians (ACCP) [15] and British Thoracic Society (BTS) criteria [16], as follows:

- ACCP definition: small pneumothorax, apex-to-cupola distance < 3 cm; large pneumothorax, distance ≥ 3 cm
- BTS definition: small pneumothorax, interpleural distance < 2 cm at the level of the hilum; large pneumothorax, distance ≥ 2 cm

In cases of disagreement, a third senior expert (with 50 years of experience) reviewed the images, and a final consensus was reached among the three reviewers.

### 2.5. Data Input for ChatGPT-5

All images were analyzed using ChatGPT-5 (OpenAI, San Francisco, CA, USA), a publicly accessible multimodal large language model as deployed via the ChatGPT interface during the initial study period (26–31 October 2025) and a follow-up evaluation on 31 December 2025.

Images were uploaded as JPEG files and interpreted independently under a fixed prompt. Prior to analysis, anonymization was implemented through removal of patient identifiers and embedded metadata. Protected health information and patient-specific clinical data were not contained in the uploaded files.

To prevent order bias and label leakage, all pneumothorax and normal CXRs were randomly renamed with sequential identifiers before being input to the ChatGPT-5. Each image was independently analyzed under the following three prompting strategies:

- Prompt A (instructional): “Whether pneumothorax is present.”
- Prompt B (role-based): Prompt A preceded by “You are an experienced pediatric radiologist.”
- Prompt C (clinical context): Prompt A preceded by “You are an experienced pediatric radiologist working in an emergency department.”

**Figure 1:**
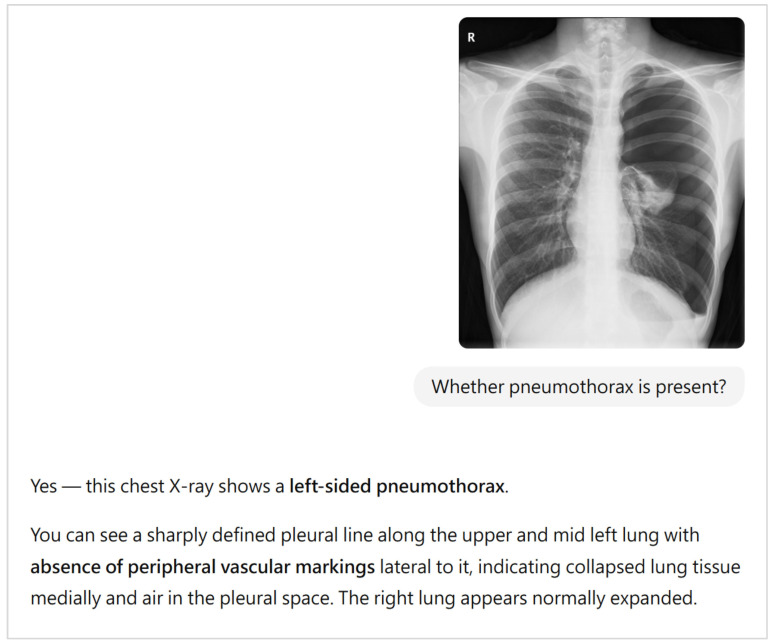
Representative example of a ChatGPT-5 question–answer interaction using Prompt A. A pediatric posteroanterior chest radiograph from a 15-year-old male patient diagnosed with pneumothorax, classified as a large pneumothorax according to expert-adjudicated reference standards based on the criteria of the American College of Chest Physicians (ACCP) and the British Thoracic Society (BTS), was uploaded to the ChatGPT interface. The model’s response exemplifies the typical output format. For the purpose of quantitative evaluation, only the presence and laterality of the pneumothorax were extracted from the model responses for analysis.

A representative ChatGPT–model question–answer exchange under Prompt A is presented in Figure 1, illustrating the workflow and model response format. Although the narrative output could include descriptive radiographic features, only pneumothorax presence and laterality were extracted for analysis in this study.

Prompt A was repeated after 48 h (A2) and 2 months later (A3, using the then-accessible ChatGPT-5.2 interface) to assess short-term reproducibility and cross-version performance stability, respectively. Each prompting session was conducted separately and within distinct chat windows to prevent model carryover and contextual memory effects. All image analyses were performed on the same laptop computer by the first author during 26–31 October and on 31 December 2025, using identical hardware and network settings to ensure reproducibility.

### 2.6. Statistical Analyses

Descriptive statistics were used to summarize the baseline characteristics of the study population. For continuous variables (e.g., age), data were expressed as medians with interquartile ranges (IQRs) due to non-normality, as determined by the Kolmogorov–Smirnov test. Between-group comparisons were performed using the Mann–Whitney U test. For categorical variables (e.g., sex), data were presented as counts and percentages, and group differences were assessed using the χ^2^ test.

Diagnostic performance of ChatGPT-5 was evaluated across the three prompting strategies using accuracy, sensitivity, and specificity. Additional performance metrics, including the area under the receiver operating characteristic curve (AUROC), positive predictive value (PPV), negative predictive value (NPV), and F1-score, are provided in the Supplementary Material (Table S1) for completeness. All metrics were computed directly from the contingency tables of ChatGPT-5’s binary outputs against the reference standard, with 95% confidence intervals (CIs) estimated using nonparametric bootstrap resampling (1000 iterations). The AUROC was derived by fitting logistic regression models to numerically coded binary outputs (1 = “present”, 0 = “absent”). In this balanced design, the resulting AUROC is mathematically equivalent to overall accuracy.

To evaluate localization performance, we reported conditional side accuracy, defined as the proportion of correct laterality assignments among true-positive cases of pneumothorax. Differences in sensitivity between small and large pneumothoraces were compared using a two-proportion z-test for independent binomial proportions.

Differences in diagnostic performance among the three prompting strategies (Prompts A–C) were evaluated using Cochran’s Q test for related proportions. Reproducibility between the initial and repeated evaluations of Prompt A (A vs. A2 and A vs. A3) was assessed using McNemar’s test for paired categorical data.

All statistical analyses were performed using IBM SPSS Statistics (version 26.0; IBM Corp., Armonk, NY, USA). A two-tailed *p*-value < 0.05 was considered statistically significant. Python (version 3.10; Python Software Foundation, Wilmington, DE, USA) was also used to verify statistical outputs and generate data visualizations.

## 3. Results

### 3.1. Study Population

A total of 380 pediatric CXRs were included in this study, comprising 190 pneumothorax cases and 190 matched controls. The two groups were well-balanced in terms of age and sex distribution, with no significant differences observed (Table 1).

### 3.2. Overall Diagnostic Performance of ChatGPT-5

Across all prompting strategies, ChatGPT-5 demonstrated moderate overall accuracy (0.77–0.79) for pneumothorax detection, with consistently high specificity (0.96–0.98) but limited sensitivity (0.57–0.61) (Table 2). The conditional side accuracy was high (>0.96), indicating precise lateral localization when the pneumothorax was correctly identified.

**Figure 2:**
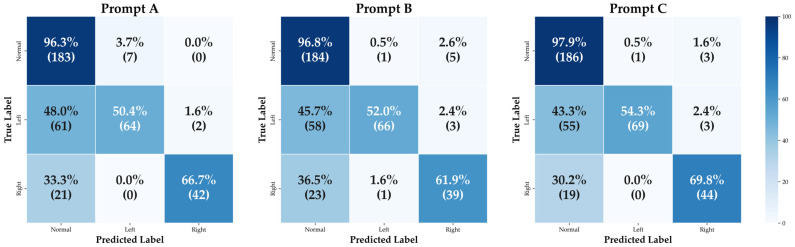
Confusion matrices illustrating ChatGPT-5’s performance in pneumothorax classification across three prompting strategies (A–C). Each cell displays the classification proportion (%) and the corresponding case count (n). While ChatGPT-5 accurately identified most normal radiographs, it misclassified 30–50% of pneumothorax cases as normal, irrespective of laterality. Among true-positive pneumothorax detections, fewer than 2.5% were assigned to the incorrect side. The clinical-context prompt (C) demonstrated a marginal improvement in detection performance compared with the instructional (A) and role-based (B) prompts.

The confusion matrix (Figure 2) illustrates this performance profile, showing that ChatGPT-5 rarely misclassified normal CXRs but missed 30–50% of pneumothorax cases. Prompt C, which embedded the clinical context, achieved marginally better performance than Prompts A and B; however, no statistically significant differences were found among the primary prompts (A–C) or between the repeated assessments of Prompt A (A vs. A2 and A vs. A3) (Table 2). Additional performance metrics, including AUROC, PPV, NPV and F1-score with 95% confidence intervals, are provided in Supplementary Table S1.

### 3.3. Impact of Pneumothorax Size on Sensitivity

**Figure 3:**
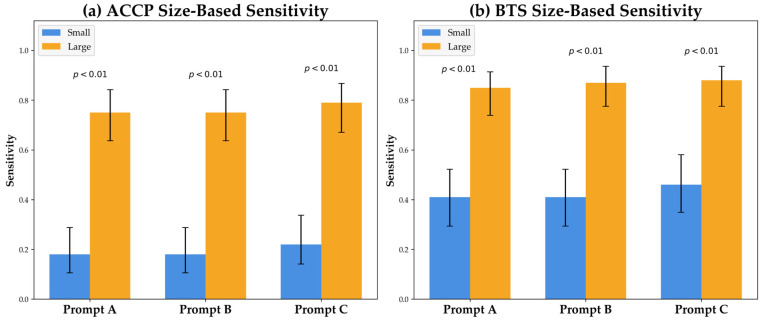
Sensitivity of ChatGPT-5 for pneumothorax detection, stratified by pneumothorax size according to the American College of Chest Physicians (ACCP) (**a**) and British Thoracic Society (BTS) (**b**) classification systems. Across all prompting strategies (A–C), sensitivity was significantly lower for small pneumothoraces (ACCP: 0.18–0.22; BTS: 0.41–0.46) than for large pneumothoraces (ACCP: 0.75–0.79; BTS: 0.85–0.88; all comparisons *p* < 0.01). Error bars represent 95% confidence intervals.

Stratified analysis revealed a strong association between pneumothorax size and ChatGPT-5 detection sensitivity, which was consistent across ACCP and BTS classification systems (Table 3, Figure 3). For small pneumothoraces, sensitivity was significantly lower (ACCP: 0.18–0.22; BTS: 0.41–0.46) compared to that for large pneumothoraces (ACCP: 0.75–0.79; BTS: 0.85–0.88) (*p* < 0.01 for all comparisons). Although no statistically significant differences were found across prompts, Prompt C consistently achieved the highest sensitivity across all size categories.

## 4. Discussion

### 4.1. Summary of Principal Findings

The present study revealed a distinct performance profile for ChatGPT-5 in detecting pediatric pneumothorax, with consistently high specificity (>0.96) and lateral localization (>0.96), but modest overall accuracy (0.77–0.79) and critically limited sensitivity (0.57–0.61). Overall, this pattern, which was consistent across prompting strategies, indicates a conservative diagnostic strategy. Most notably, model performance was substantially worse for small pneumothoraces, underscoring its current suboptimal capability for identifying subtle radiographic signs in pediatric patients.

### 4.2. Comparison with Prior Multimodal LLM Studies

Recent studies have increasingly explored the potential of multimodal LLMs in chest imaging diagnoses; however, their reported performances are variable. For instance, Lacaita et al. reported that ChatGPT-4o achieved an overall accuracy of 69% for CXRs and abdominal radiographs, with slightly lower performance for CXRs (66%), and with only approximately 41% accuracy in pneumothorax detection. These findings suggest that although the model is likely capable of recognizing conspicuous pathologies such as pulmonary edema or pneumonia, its sensitivity to subtle or apical pneumothoraces remains limited [17]. Bulut et al. compared three multimodal LLMs—ChatGPT-4o, Gemini 2.0, and Claude 3.5—and observed notable discrepancies across age groups and pneumothorax sizes. ChatGPT-4o achieved relatively high accuracy for large pneumothoraces in adults (approximately 82%), but its accuracy declined sharply to approximately 20–42% in pediatric or small pneumothorax cases [18]. Although their work provided pivotal insights into age-related performance decline, the evaluation was constrained by the use of a single non-varying prompt and the absence of a control group. To systematically investigate the impact of these methodological factors, we evaluated multiple prompting strategies, including instructional, role-based, and clinical context formulations, and incorporated a matched control group of normal CXRs. This design allowed us to assess the influence of linguistic framing on diagnostic behavior and enabled a robust estimation of specificity and sensitivity. The consistent trend of higher sensitivity with the emergency department–context prompt (Prompt C), while statistically insignificant, suggests that embedding the clinical context might modestly guide the reasoning pattern of the LLM by potentially lowering its detection threshold.

Ostrovsky et al. employed a distinct approach by integrating ChatGPT-4.0 within the “X-Ray Interpreter” GPT add-on. This configuration constrains the model to analyze images without additional clinical history, guiding it through a predefined structured analysis of specific anatomical regions (e.g., lung fields, mediastinum, and osseous structures). Although this integration of visual and linguistic reasoning yielded a pneumothorax sensitivity of 77.4%, the diagnostic accuracy was noted to remain below clinically acceptable thresholds, particularly for more subtle pathologies [19].

In contrast to previous investigations that primarily used adult or mixed-age datasets, the present study focused exclusively on a pediatric cohort. The study design enabled specific insights into the real-world feasibility of LLM-based pneumothorax detection in this population. Furthermore, our evaluation assessed not only the presence of pneumothorax but also its laterality and size-based severity, allowing for a more granular analysis of the model’s localization performance.

Collectively, previous studies and the present findings converge on a common conclusion: despite their accessibility and incremental progress in multimodal reasoning, the ability of current LLMs to detect subtle thoracic abnormalities, particularly in pediatric and small pneumothorax cases, remains insufficient to support autonomous clinical deployment.

### 4.3. Comparison with Specialized AI Models

To contextualize the performance of LLMs, we briefly review established specialized models such as CNNs, which have demonstrated promising performance in CXR interpretation [8,9,20]. Early deep-learning architectures such as DenseNet121 and customized MobileNetV2 derivatives achieved excellent accuracy when trained on large-scale labeled datasets such as ChestX-ray14. For instance, the fine-tuned MobileLungNetV2 model reported an overall classification accuracy of 96.97% with precision, recall, and specificity all exceeding 96%, highlighting the maturity of supervised CNN frameworks for thoracic image analysis [21].

Commercial CNN tools have demonstrated comparable robustness in real-world validation studies. van Beek et al. evaluated a machine-learning software (Lunit INSIGHT CXR, version 3.1.2.0) across both primary care and emergency department settings, demonstrating AUROC values between 0.88 and 0.99 for major thoracic findings, including pneumothorax, pleural effusion, and airspace disease [22]. However, Plesner et al. conducted a large-scale assessment of multiple commercially available CXR AI products and reported that detection sensitivity often decreased as the target lesion, such as a pneumothorax or parenchymal opacity, became smaller [23]. This established limitation of specialized CNNs was clearly echoed in our ChatGPT-5 evaluation, which exhibited a similar decrease in sensitivity for smaller pneumothoraces. This recurrence of size-dependent performance decay across fundamentally different architectures indicates a common underlying bottleneck, which is the intrinsic difficulty in interpreting subtle visual representations of small- or low-contrast findings.

### 4.4. Toward Hybrid Integration of LLM and CNN Frameworks

Convolutional neural network (CNN)–based models have been widely used in the domain of medical image analysis. These models are specifically engineered to process grid-like pixel data while effectively capturing hierarchical spatial features. This architectural design facilitates explicit localization and visualization techniques, such as Grad-CAM or probability heat maps, which serve to highlight image regions that contribute to classification decisions [5,9,21].

In contrast, multimodal large language models (LLMs) such as ChatGPT-4o and ChatGPT-5 are inherently designed for semantic comprehension and language-based reasoning, rather than for the precise extraction of pixel-level coordinates. These models interpret radiographs through the semantic alignment of visual and textual embeddings, enabling them to reason across modalities and integrate radiographic patterns within a linguistic and clinical context to produce coherent diagnostic interpretations [19,24].

In light of these architectural differences, the current study deliberately concentrated on evaluating the semantic diagnostic outputs of ChatGPT-5, specifically pneumothorax presence and laterality. This design choice reflects a pragmatic approach to assessing the diagnostic behavior of multimodal LLMs at their present stage of development and is aligned with previous studies on pneumothorax using ChatGPT [17,18].

Consistent with this design, Huang et al. demonstrated that generative transformer-based AI could generate emergency department CXR reports with a diagnostic accuracy comparable to that of in-house radiologists, while surpassing teleradiology in narrative coherence [25]. Chang et al. recently introduced a hybrid curriculum learning framework in which ChatGPT was used to stratify pneumothorax cases according to difficulty, enabling a deep learning model (EfficientNetB3) to achieve near-Food and Drug Administration (FDA)-grade performance (AUROC = 0.98) across multiple institutions [7]. Their work exemplifies how LLM-assisted preprocessing can enhance model performance in challenging cases such as small lesions. This aligns at the conceptual level with our use of LLM-assisted prompting. Both approaches leverage the model’s semantic reasoning to improve outcomes when pure visual analysis falls short.

Taken together, these findings suggest that LLMs are probably best positioned as cognitive integrators rather than standalone detectors capable of harmonizing linguistic contexts with radiologic patterns. A hybrid CNN–LLM paradigm that couples pixel-level perception with contextual reasoning is likely to represent the next stage of clinical AI development, potentially improving the interpretability, robustness, and workflow adaptability in complex diagnostic environments.

### 4.5. Limitations

This study had several limitations. First, its retrospective, single-center design may have introduced a selection bias and limited generalizability. The uneven distribution of the pneumothorax laterality and size in our cohort may have contested the objective evaluation of the diagnostic performance of the model across these subgroups.

Second, the analysis was confined to a single pathology (pneumothorax) and single radiographic projection (PA view), with the deliberate exclusion of cases involving concomitant pulmonary disease, indwelling chest tubes, and imaging artifacts. Although this approach allowed for a controlled evaluation, it resulted in a “clean” dataset that does not fully represent the conditions encountered in real-world emergency departments, where overlapping pathologies, medical devices, and suboptimal imaging are common. Previous studies have indicated that the diagnostic performance of AI systems may be inferior on anteroposterior views compared to PA views, with structures such as skin folds frequently causing false-positive pneumothorax detections [23]. Consequently, the specificity and real-world applicability of the model in mixed clinical scenarios remain uncertain.

Third, the study population had a relatively older pediatric age distribution (median 16.8 years), and the findings should be extrapolated to younger children with caution. Younger children are characterized by marked differences in thoracic anatomy, including chest-wall configuration, mediastinal contours, and rib-cage size and density [10]. In addition, greater chest-wall compliance and smaller lung volumes in younger patients can be associated with less conspicuous pleural separation in pneumothorax, thereby increasing diagnostic difficulty [18]. Collectively, these age-related anatomical differences could constrain the generalizability of our findings to younger pediatric populations.

Fourth, expert consensus was used as the reference standard rather than independent clinician reads. Consequently, a direct comparison between ChatGPT-5 and human readers across experience levels in clinical practice was not enabled. Future studies incorporating head-to-head human–AI comparisons will be necessary to contextualize the clinical relevance of multimodal LLMs.

Fifth, all chest radiographs were analyzed as JPEG files, without access to window-level adjustment. Because windowing is frequently used to increase the conspicuity of subtle pleural findings, this constraint could have reduced the detectability of small pneumothoraces and contributed to lower sensitivity [12].

Sixth, given the continuously updated nature of deployed LLMs, our evaluation represents a time-stamped assessment of ChatGPT performance during the study period. While no significant differences were observed between ChatGPT-5 and ChatGPT-5.2 using Prompt A, future platform updates may influence diagnostic behavior and should be considered when interpreting these findings.

Finally, as a closed “black-box” system, ChatGPT-5 precludes the direct assessment of its internal reasoning or visual attention processes. Accordingly, detailed characterization of false-negative cases by lesion localization, subtlety, or image exposure was not feasible, limiting mechanistic interpretation of the observed low sensitivity and constraining identification of model-improvement priorities. Moreover, such systems remain capable of generating factually incorrect information (hallucinations) [26,27].

Furthermore, it is important to note that ChatGPT and related LLMs currently lack regulatory certification as medical devices (e.g., FDA clearance or Conformité Européenne marking), which precludes their use as primary diagnostic tools in clinical practice.

## 5. Conclusions

In summary, ChatGPT-5 demonstrated consistent yet limited diagnostic performance for pediatric pneumothorax on CXRs. Its high specificity and reproducible detection of larger pneumothoraces reflect favorable performance characteristics. However, its unacceptably low sensitivity for subtle cases precludes its standalone clinical use and necessitates further optimization.

## Figures and Tables

**Table 1:** Baseline Characteristics of the Pediatric Chest Radiograph Cohort.

Characteristic	Control (n = 190)	Pneumothorax (n = 190)	*p*-Value
Age, years	16.8 (16.2–17.3)	16.8 (16.0–17.5)	0.41 ^†^
Male, No. (%)	170 (89.5)	170 (89.5)	>0.99 ^‡^
Pneumothorax Laterality, No. (%)			
Left	N/A	127 (66.8)	
Right	N/A	63 (33.2)	
Pneumothorax Size (ACCP), No. (%)			
Small (<3 cm)	N/A	60 (31.6)	
Large (≥3 cm)	N/A	130 (68.4)	
Pneumothorax Size (BTS), No. (%)			
Small (<2 cm)	N/A	122 (64.2)	
Large (≥2 cm)	N/A	68 (35.8)	

Notes: Data are presented as median (interquartile range [IQR]) for age and as number (percentage [%]) for categorical variables. The control group was age- and sex-matched. Abbreviation: ACCP, American College of Chest Physicians; BTS, British Thoracic Society; N/A, Not applicable. Statistical tests: ^†^ Mann–Whitney U test. ^‡^ χ^2^ test.

**Table 2:** Diagnostic Performance of ChatGPT-5 for Pneumothorax Detection Across Prompting Strategies.

Prompt	Accuracy	Sensitivity	Specificity	Conditional Side Accuracy *
A	0.77 (0.72–0.81)	0.57 (0.49–0.64)	0.96 (0.93–0.99)	0.98 (0.93–1.00)
B	0.77 (0.73–0.81)	0.57 (0.50–0.65)	0.97 (0.93–0.99)	0.96 (0.91–0.99)
C	0.79 (0.75–0.83)	0.61 (0.54–0.68)	0.98 (0.95–0.99)	0.97 (0.93–0.99)
A2	0.79 (0.74–0.83)	0.61 (0.53–0.68)	0.97 (0.93–0.99)	0.97 (0.93–0.99)
A3	0.77 (0.73–0.81)	0.58 (0.51–0.65)	0.96 (0.92–0.98)	0.98 (0.94–1.00)

Notes: Data represent the value (95% confidence interval). Prompt A: instructional; Prompt B: role-based; Prompt C: clinical-context; Prompt A2: 48 h repeat of Prompt A; Prompt A3: 2-month repeat of Prompt A using ChatGPT-5.2. No statistically significant differences were observed among Prompts A–C (Cochran’s Q = 0.98, *p* = 0.613) or between A and A2 (McNemar’s χ^2^ = 0.78, *p* = 0.377), or between A and A3 (McNemar’s χ^2^ = 0.35, *p* = 0.556). * Conditional side accuracy is defined as the proportion of correct laterality assignments among true-positive cases.

**Table 3:** Sensitivity of ChatGPT-5 for Pneumothorax Detection by Size and Classification System.

Prompt	System	Size	Sensitivity (95% CI)
A	ACCP	Small	0.18 (0.10–0.30)
		Large	0.75 (0.66–0.82)
	BTS	Small	0.41 (0.33–0.50)
		Large	0.85 (0.75–0.93)
B	ACCP	Small	0.18 (0.10–0.30)
		Large	0.75 (0.67–0.83)
	BTS	Small	0.41 (0.32–0.50)
		Large	0.87 (0.76–0.94)
C	ACCP	Small	0.22 (0.12–0.34)
		Large	0.79 (0.71–0.86)
	BTS	Small	0.46 (0.37–0.55)
		Large	0.88 (0.78–0.95)

Notes: Sensitivity was significantly higher for large pneumothoraces than for small pneumothoraces (two-proportion z-test, all *p* < 0.01). No statistically significant differences were observed across prompting strategies. Abbreviation: ACCP, American College of Chest Physicians; BTS, British Thoracic Society.

## Data Availability

The datasets generated and analyzed during the current study are not publicly available due to patient privacy regulations and the data governance policies of MacKay Memorial Hospital. Requests for access to de-identified data from qualified researchers may be submitted to the corresponding author and will require a formal data use agreement and approval from the MacKay Memorial Hospital Institutional Review Board.

## Supplementary Materials

The following supporting information can be downloaded at: https://www.mdpi.com/article/10.3390/diagnostics16020232/s1, Table S1: Performance metrics of ChatGPT-5 for pneumothorax detection across different prompting strategies (AUROC, PPV, NPV, and F1-score), with 95% CIs derived from non-parametric bootstrap resampling (1000 iterations). Prompt A: instructional; Prompt B: role-based; Prompt C: clinical-context; Prompt A2: 48-h repeat of Prompt A; Prompt A3: 2-month repeat of Prompt A using ChatGPT-5.2.

## Abbreviations

The following abbreviations are used in this manuscript:

AI	Artificial Intelligence
CNN	Convolutional Neural Network
LLM	Large Language Model
STROBE	Strengthening the Reporting of Observational Studies in Epidemiology
CXR	Chest X-ray
EMR	Electronic Medical Record
PACS	Picture Archiving and Communication System
ICD	International Classification of Diseases
PA	Posterior–anterior
IQR	Interquartile Range
ACCP	American College of Chest Physicians
BTS	British Thoracic Society
CI	Confidence Interval
AUROC	Area under the Receiver Operating Characteristic
FDA	Food and Drug Administration
PPV	Positive Predictive Value
NPV	Negative Predictive Value
