# Supplementary material for: Diagnostic Performance of ChatGPT-5 for Detecting Pediatric Pneumothorax on Chest Radiographs: A Multi-Prompt Evaluation
Source: Diagnostics (Basel). 2026 Jan 11;16(2):232. doi: 10.3390/diagnostics16020232 (PMC12839758; doi:10.3390/diagnostics16020232)
Supplement: Supplementary file 1 [file diagnostics-16-00232-s001.zip › diagnostics-4054499-supplementary.pdf]

Table S1. Performance Metrics of ChatGPT-5 for Pneumothorax Detection Across Prompting Strategies

Prompt	AUROC	PPV	NPV	F1-score
**A**	0.77 (0.73–0.80)	0.94 (0.89-0.98)	0.69 (0.64-0.75)	0.71 (0.65-0.76)
**B**	0.77 (0.73–0.81)	0.95 (0.90-0.98)	0.69 (0.64-0.75)	0.71 (0.65-0.77)
**C**	0.79 (0.76–0.83)	0.97 (0.93-0.99)	0.72 (0.66-0.77)	0.75 (0.70-0.80)
**A2**	0.79 (0.75–0.82)	0.95 (0.91-0.98)	0.71 (0.66-0.76)	0.74 (0.68-0.79)
**A3**	0.77 (0.73–0.81)	0.94 (0.88-0.97)	0.70 (0.64-0.75)	0.72 (0.66-0.77)

Notes: Data are shown as values with 95% confidence intervals. Confidence intervals were derived using non-parametric bootstrap resampling (1,000 iterations). AUROC values reflect single-threshold performance and are mathematically equivalent to overall accuracy.

Prompt A: instructional; Prompt B: role-based; Prompt C: clinical-context; Prompt A2: 48-hour repeat of Prompt A; Prompt A3: 2-month repeat of Prompt A using ChatGPT-5.2. Abbreviations: AUROC, Area under the Receiver Operating Characteristic; PPV, Positive Predictive Value; NPV, Negative Predictive Value.
